# Novos Biomarcadores Cardiovasculares em Pacientes com Câncer de Mama Submetidas a Quimioterapia à Base de Doxorrubicina

**DOI:** 10.36660/abc.20230167

**Published:** 2023-12-14

**Authors:** Rodrigo Mendonça Cardoso Pestana, Júnea Paolucci Paiva Silvino, Angélica Navarro de Oliveira, Cintia Esteves Soares, Adriano de Paula Sabino, Ricardo Simões, Karina Braga Gomes

**Affiliations:** 1 Faculdade de Medicina Universidade Federal de Minas Gerais Belo Horizonte MG Brasil Faculdade de Medicina – Universidade Federal de Minas Gerais , Belo Horizonte , MG – Brasil; 2 Instituto de Hipertensão Belo Horizonte MG Brasil Instituto de Hipertensão , Belo Horizonte , MG – Brasil; 3 Fundação Hospitalar do Estado de Minas Gerais Belo Horizonte MG Brasil Fundação Hospitalar do Estado de Minas Gerais (FHEMIG), Belo Horizonte , MG – Brasil; 4 Faculdade de Farmácia Universidade Federal de Minas Gerais Belo Horizonte MG Brasil Faculdade de Farmácia - Universidade Federal de Minas Gerais , Belo Horizonte , MG – Brasil

**Keywords:** Neoplasias da Mama, Doenças Cardiovasculares, Doxorrubicina, Biomarcadores

## Abstract

**Fundamento:**

As doenças cardiovasculares (DCV) são relevantes para o manejo do tratamento do câncer de mama, uma vez que um número significativo de pacientes desenvolve essas complicações após a quimioterapia.

**Objetivo:**

Este estudo teve como objetivo avaliar novos biomarcadores cardiovasculares, sendo eles CXCL-16 (ligante de motivo C-X-C 16), FABP3 (proteína de ligação a ácidos graxos 3), FABP4 (proteína de ligação a ácidos graxos 4), LIGHT (membro da superfamília do fator de necrose tumoral 14/TNFS14), GDF-15 (fator de crescimento/diferenciação 15) , sCD4 (forma solúvel de CD14) e ucMGP (matriz Gla-proteína não carboxilada) em pacientes com câncer de mama tratadas com doxorrubicina (DOXO).

**Métodos:**

Este estudo de caso-controle foi realizado em uma clínica oncológica, incluindo 34 mulheres com diagnóstico de câncer de mama tratadas com quimioterapia com DOXO e 34 mulheres controle, sem câncer ou DCV. Os marcadores foram determinados imediatamente após o último ciclo de quimioterapia. O nível de significância estatística adotado foi de 5%.

**Resultados:**

O grupo com câncer de mama apresentou níveis mais elevados de GDF-15 (p<0,001), enquanto os indivíduos controle apresentaram níveis mais elevados de FABP3 (p=0,038), FABP4 (p=0003), sCD14 e ucMGP (p<0,001 para ambos). Correlações positivas foram observadas entre FABPs e IMC no grupo com câncer.

**Conclusão:**

GDF15 é um biomarcador emergente com potencial aplicabilidade clínica neste cenário. FABPs são proteínas relacionadas à adiposidade, potencialmente envolvidas na biologia do câncer de mama. sCD14 e ucMGP estão envolvidos na calcificação inflamatória e vascular. Acima de tudo, a avaliação destes novos biomarcadores cardiovasculares pode ser útil no tratamento da quimioterapia do câncer de mama com DOXO.

## Graphical abstract

**Figura Central:**
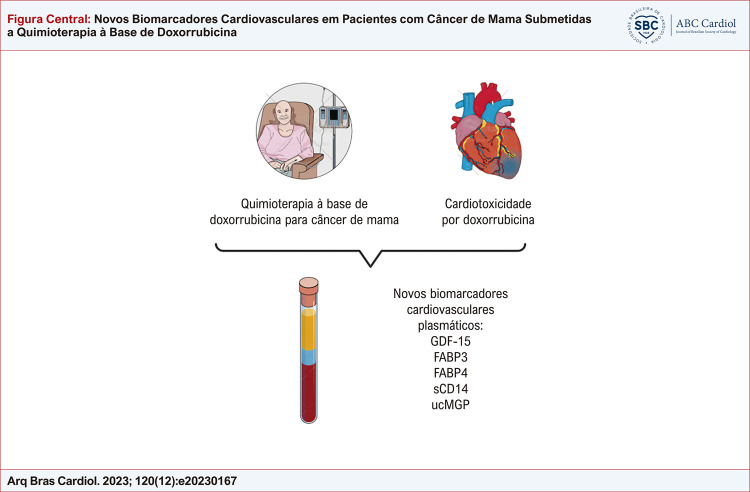
: Novos Biomarcadores Cardiovasculares em Pacientes com Câncer de Mama Submetidas a Quimioterapia à Base de Doxorrubicina

## Introdução

As doenças cardiovasculares (DCV) e o câncer são as principais causas de morte em todo o mundo. ^1^ Melhorias contínuas nas estratégias de prevenção e tratamentos anticâncer em pacientes com câncer de mama reduziram significativamente o número de mortes por causas relacionadas à doença; no entanto, houve um risco aumentado de morte por DCV neste grupo de pacientes. ^2^ As razões para este sinergismo entre câncer e as DC são os fatores que ambos possuem em comum (incluindo diabetes mellitus, hipertensão, hipercolesterolemia e obesidade), bem como os mecanismos fisiopatológicos subjacentes às DCV, associados a um risco aumentado de câncer. ^3 , 4^

Os regimes de tratamento baseados em antraciclinas, como a doxorrubicina (DOXO), são algumas das alternativas mais eficazes do câncer da mama, sendo responsáveis pela melhoria da sobrevida livre de doença e da sobrevida global neste grupo. No entanto, as antraciclinas podem resultar em toxicidade grave a curto e longo prazo, incluindo cardiotoxicidade e malignidade hematológica secundária. ^5^

Diversos estudos propuseram o uso de biomarcadores plasmáticos, especialmente troponinas e peptídeos natriuréticos tipo B, para monitorar a cardiotoxicidade das antraciclinas visando a detecção precoce dessas complicações cardiovasculares. ^6 - 8^ Mais recentemente, esses biomarcadores foram incluídos como critérios diagnósticos de cardiotoxicidade, além dos exames de imagem cardiológicos e suas modalidades e características clínicas. ^9 , 10^ Outros biomarcadores são subjacentes às alterações fisiopatológicas que ocorrem durante a insuficiência cardíaca. A insuficiência cardíaca se manifesta como diminuição da fração de ejeção do ventrículo esquerdo (FEVE) ou insuficiência cardíaca sintomática em até 5% dos pacientes. ^11^ Em um estudo prospectivo, Cardinale et al. ^12^ observaram uma incidência global de cardiotoxicidade de 9% usando a diminuição da FEVE como critério único para definição de cardiotoxicidade. Porém, em outro estudo prospectivo, López-Sendón et al. ^10^ ampliaram os critérios de definição de cardiotoxicidade para além das alterações da FEVE, incluindo o uso de biomarcadores plasmáticos e cardiotoxicidade, que foi identificada em 37,5% dos pacientes durante o acompanhamento.

As quimiocinas são citocinas quimioatrativas pró-inflamatórias que atuam principalmente no tráfego de leucócitos, regulam a migração, proliferação e sobrevida celular e são componentes-chave na biologia do câncer. ^13^ A CXCL-16 (ligante do motivo C-X-C 16) é uma quimiocina expressa em órgãos linfoides, fígado, pulmões, intestino delgado e rim. A expressão de CXCL-16 é aumentada por citocinas pró-inflamatórias, importantes para o acúmulo de células imunes nos locais de reação inflamatória. ^14^

O LIGHT (membro da superfamília do fator de necrose tumoral 14/TNFS14) pertence à superfamília do fator de necrose tumoral e é expresso por diferentes tipos de células do sistema imunológico. O LIGHT sinaliza através de dois receptores e possui funções distintas que dependem do tipo de célula, mas as interações com esses tipos de receptores têm implicações imunológicas na biologia do tumor. ^15 , 16^

O fator de crescimento/diferenciação 15 (GDF-15) é um membro divergente da superfamília do fator de crescimento transformador-β (TGF-β), sendo também conhecido como citocina inibitória de macrófagos (MIC)-1. ^17^ O GDF-15 também está relacionado com a evolução do câncer, tanto positiva quanto negativamente, uma vez que inibe a promoção tumoral precoce, mas sua expressão anormal em cânceres avançados causa formação de células-tronco cancerígenas, proliferação, invasão, metástase, escape imunológico e uma resposta reduzida à terapia. ^18^

A proteína matriz-carboxiglutamato (Gla) (MGP) é uma proteína dependente de vitamina K e um forte inibidor da calcificação vascular. A deficiência de vitamina K leva à MGP não carboxilada (ucMGP) inativa, que se acumula em locais de calcificação arterial. ^19^ Biologicamente inativo, o desfosfo-ucMGP é um marcador da condição da vitamina K vascular, sendo descrito como preditor de mortalidade em pacientes com insuficiência cardíaca e estenose aórtica. ^20^

O antígeno de diferenciação de monócitos humanos CD14 é um receptor de reconhecimento de padrões (RRP) que aumenta as respostas imunes inatas. O CD14 foi identificado pela primeira vez como um marcador de monócitos para sinalizar respostas intracelulares após encontros bacterianos. ^21^ Formas solúveis de CD14 (sCD14) podem ser secretadas por células ativadas, que liberam CD14 por eliminação dependente ou independente de proteinase. ^22^

As FABPs (proteínas de ligação a ácidos graxos) são proteínas expressas em quase todos os tecidos. Essas proteínas são responsáveis pelo controle do transporte, metabolismo e armazenamento de ácidos graxos. As FABPs são propostas como reguladoras centrais do metabolismo lipídico, da inflamação e da homeostase energética. ^23^ A FABP3 é uma proteína citosólica encontrada principalmente no coração, mas também nos músculos, cérebro e rins. ^24^ Alguns estudos sugerem que a FABP3 tem sensibilidade superior à troponina para detecção de lesão isquêmica e lesão cardíaca associada à insuficiência cardíaca congestiva. ^25 , 26^ A FABP4 é expressa principalmente em adipócitos e macrófagos e desempenha um papel importante no desenvolvimento da resistência à insulina e aterosclerose. Os níveis circulantes de FABP4 estão associados a diversos aspectos da síndrome metabólica e doenças cardiovasculares. ^27^

A implementação de novos biomarcadores laboratoriais tem sido uma prioridade na cardio-oncologia, principalmente para a detecção precoce de cardiotoxicidade secundária à quimioterapia. Nesse contexto, este estudo teve como objetivo avaliar novos biomarcadores cardiovasculares, como CXCL-16, FABP4, LIGHT, GDF-15, sCD14 e ucMGP em pacientes com câncer de mama sob quimioterapia baseada em DOXO.

## Participantes e Métodos

### Amostras humanas

Trata-se de um estudo caso-controle realizado com pacientes ambulatoriais do Serviço de Oncologia do Hospital Alberto Cavalcanti/FHEMIG (Belo Horizonte, Brasil), que incluiu 34 mulheres com idade igual ou superior a 18 anos com diagnóstico de câncer de mama e em uso de terapia neoadjuvante com DOXO, atendidas no período entre junho de 2015 e junho de 2018. Os critérios de exclusão no grupo caso foram: presença de cardiopatia prévia com função ventricular esquerda prejudicada; disfunção hepática ou renal moderada a grave; doenças cerebrais degenerativas que necessitem de cuidadores; e gestantes ou pacientes com expectativa de vida inferior a três meses. Além disso, mulheres que já haviam sido submetidas a quimioterapia, terapia hormonal, imunoterapia ou radioterapia foram excluídas. O grupo controle foi formado por 34 mulheres saudáveis com idade igual ou superior a 18 anos, sem qualquer doença maligna ou presença de cardiopatia prévia, disfunção hepática ou renal moderada a grave, doenças degenerativas e não gestantes, conforme atestado por médico clínico.

As características clínicas dos pacientes com câncer de mama foram obtidas a partir dos prontuários médicos hospitalares. Antes da quimioterapia, as pacientes com câncer de mama foram submetidas a avaliação médica com um cardiologista, que realizou eletrocardiograma e ecocardiograma bidimensional incluindo modo tecidual com ecocardiografia usando o Vivid S6 (GE Medical Systems Healthcare®, Tirat Carmel, Israel) com avaliação da FEVE. Não foram observadas alterações nesses exames. O risco cardiovascular de pacientes com câncer de mama foi calculado de acordo com o escore de risco global ( *Framingham Heart Study* ) ^28^ antes do tratamento do câncer.

O estudo foi aprovado pelo Comitê de Ética em Pesquisa da UFMG (n.º 38538714.20000.5149) e Comitê de Ética da FHEMIG (n.º 54376216.0.0000.5119), seguindo os preceitos da Declaração de Helsinque da Associação Médica Mundial. Todas as participantes assinaram previamente o termo de consentimento livre e esclarecido.

### Protocolos experimentais e laboratoriais

A coleta de sangue em jejum foi realizada após a quimioterapia à base de DOXO (até sete dias após o último ciclo de DOXO). Para o preparo do plasma, o tubo de EDTA foi centrifugado por 10 minutos a 1000 g dentro de 30 minutos após a coleta do sangue e para o preparo do soro, o tubo sem aditivo foi centrifugado a 3000 g por 15 min. As amostras de plasma e soro foram distribuídas em alíquotas e imediatamente armazenadas a -80°C até a análise.

Os níveis dos marcadores cardiovasculares foram determinados por imunoensaios multiplexados utilizando a plataforma Luminex® xMAP®. O plasma de EDTA foi utilizado para determinações de CXCL-16, FABP3, FABP4, LIGHT (kit HCVD1MAG-67K; Merck®, Darmstadt, Alemanha), GDF-15 (kit HCVD2MAG-67K; Merck®, Darmstadt, Alemanha), sCD14 e ucMGP (kit HCVD6MAG-67K; Merck®, Darmstadt, Alemanha), de acordo com as instruções do fabricante em um dispositivo MAGPIX® Multiplexing System Analyzer (Luminex Corporation®, Austin, EUA).

Os níveis de cTnI (troponina I) e NT-proBNP (fração NT do peptídeo natriurético tipo B), bem como a FEVE, para monitorar a avaliação da disfunção cardíaca, foram determinados de acordo com os protocolos descritos em estudo anterior. ^29^ A análise do colesterol total e do HDL foi realizada por dosagem colorimétrica no aparelho VITROS 5600 (Ortho Clinical Diagnostics®, Rochester, EUA). O colesterol LDL foi calculado pela fórmula de Friedwald.

### Análise estatística

Os dados foram analisados por meio do software IBM® SPSS® Statistics (para Windows®; Chicago, Illinois, EUA, versão 21). O teste de Shapiro-Wilk foi utilizado para verificar a normalidade das variáveis quantitativas, que foram apresentadas como média ± desvio padrão (DP) ou mediana (percentis 25–75). O teste t de Student não pareado e o ANOVA One-Way (seguido do teste de Tukey) ou o teste de Mann-Whitney e Kruskal-Wallis (seguido do teste de Bonferroni) determinaram as diferenças entre dois e três grupos, conforme apropriado. As variáveis categóricas foram apresentadas como n (%) e comparadas por meio do teste exato de Fisher. As correlações foram realizadas por meio do teste de correlação de Spearman. Curvas de características de operação do receptor (ROC) foram utilizadas para representar a sensibilidade e a especificidade. O nível de significância adotado foi de 5%.

## Resultados

As médias de idade e índice de massa corporal (IMC) dos pacientes com câncer de mama antes da quimioterapia e dos controles são apresentados na Tabela 1 . Não foi observada diferença entre os grupos. Em relação à hipertensão arterial e diabetes mellitus, o grupo com câncer de mama apresentou frequências maiores que o grupo controle (todos p<0,05). Após o tratamento, todas as pacientes com câncer de mama apresentaram função ventricular normal (FEVE ≥50%). Onze (32,3%) pacientes apresentaram níveis de NT-proBNP acima do valor de referência (<125,0 pg/mL se <75 anos, ou <450,0 pg/mL, se ≥75 anos), mas nenhuma alteração nos níveis de cTnI (faixa normal <0,120 ng/mL) foi encontrado no grupo de câncer de mama. Nenhuma paciente apresentou cardiotoxicidade clínica. Outras características das pacientes com câncer de mama estão resumidas na Tabela 1 .

**Tabela 1:** – Características clínicas de pacientes com câncer de mama antes da quimioterapia baseada em DOXO e controles

Variável	Câncer de mama (n=34)	Controle (n=34)	Valor de p
**Idade (anos)**	50,2 ± 11,3	46,9 ± 16,9	0,349
**IMC (kg/m** ^**2**^ **)**	27,91 ± 5,9	26,5 ± 6,2	0,340
**Diabetes mellitus, sim (n, %)**	3 (8.8)	2 (5.8)	<0,001*
**Hipertensão arterial, sim (n, %)**	11 (32.4)	9 (26.8)	0,008*
**Dose de DOXO (mg/m** ^**2**^ **)**	380,6 [360,0 – 400,0]	-	-
**FEVE (%)**	66,97 ± 2,33	-	-
**Diagnóstico histológico, n (%)**			
Carcinoma ductal invasivo	30 (88.2)		
Carcinoma lobular	3 (8.8)		
Tipos especiais	1 (2.9)	-	-
**Tipo molecular**			
HER2+	18 (52.9)		
Luminal	12 (35.3)		
Triplo negativo	4 (11.8)	-	-

IMC: índice de massa corporal; DOXO: doxorrubicina; FEVE: fração de ejeção do ventrículo esquerdo. *Valor de p significativo < 0,050.

**Figura 1:**
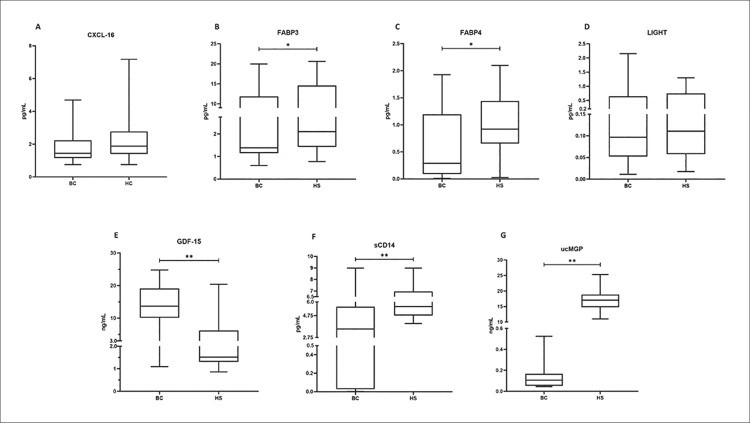
– Marcadores cardiovasculares comparando pacientes com câncer de mama e grupo controle. CXCL-16: Ligante do motivo C-X-C 16; FABP3: proteína de ligação a ácidos graxos 3; FABP4: proteína de ligação a ácidos graxos 4; LIGHT: membro da superfamília do fator de necrose tumoral 14/TNFS14; GDF-15: Fator de crescimento/diferenciação 15; sCD4: forma solúvel de CD14; ucMGP: Proteína Gla de matriz não carboxilada; BC: pacientes com câncer de mama; HS: controles saudáveis * Significativo (p<0,05). ** Significativo (p<0,001).

**Figura 2:**
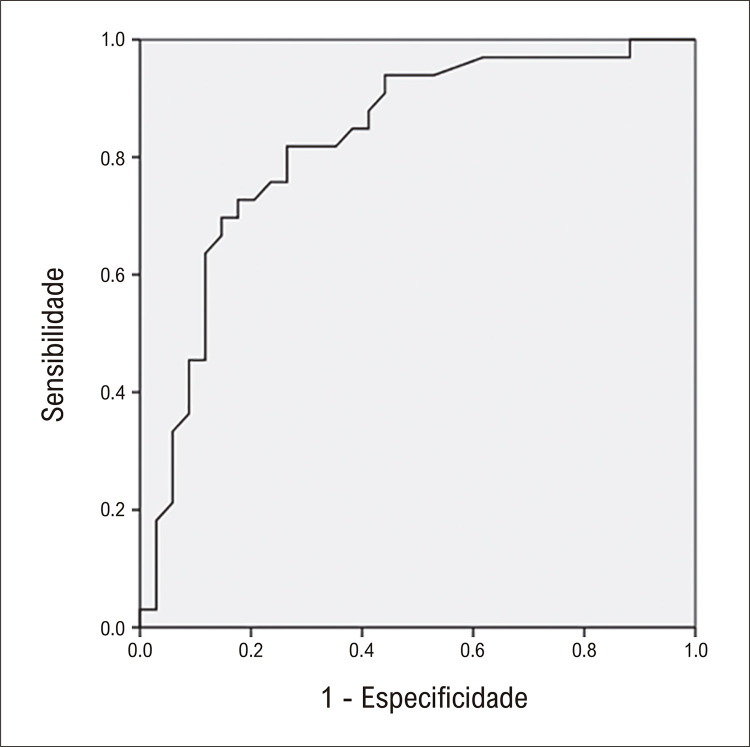
– Curva de COR para níveis de GDF-15, considerando pacientes com câncer de mama tratados com doxorrubicina x grupo controle.

A comparação dos marcadores cardiovasculares entre o câncer de mama e o grupo controle está descrita na Figura 1 . Pacientes com câncer de mama apresentaram níveis plasmáticos mais elevados de GDF-15 (p<0,001) e níveis mais baixos de FABP3 (p=0,038), FABP4 (p=0,003), sCD14 e ucMGP (p<0,001) em comparação com o grupo controle. Para o GDF-15 observou-se área sob a curva de COR =0,825 (p<0,001, IC=0,722-0,927) ( Figura 2 ). FABP3 (r=0,344; p=0,046) e FABP4 (r=0,479; p=0,004) correlacionaram-se positivamente com o IMC no grupo com câncer de mama.

Considerando o grupo com câncer de mama, é interessante notar que os níveis de GDF-15 foram maiores no grupo triplo negativo em comparação com os demais grupos moleculares (p=0,030), mas essa diferença não foi significativa após a aplicação do teste de Bonferroni ( Tabela 2 ). Os níveis de FABP3 também foram maiores no grupo com alto risco cardiovascular de Framingham (p=0,022), mas não foram significativos após a correção de Bonferroni ( Tabela 3 ). Nenhum outro marcador cardiovascular apresentou diferenças nos níveis plasmáticos de acordo com o tipo molecular do tumor ou risco cardiovascular.

**Tabela 2:** – Biomarcadores cardiovasculares de acordo com o subtipo molecular em pacientes com câncer de mama

Biomarcadores	Luminal (12, 35,3%)	HER2+ (18; 52,9%)	Triplo negativo (4; 11,8%)	Valor de p
CXCL-16 (pg/mL)	1,35 [0,96 - 1,59]	1,52 [1,25 - 2,53]	1,38 [1,03 - 2,52]	0,370
FABP3 (pg/mL)	1,36 [1,15 - 12,89]	1,21 [0,92 - 1,80]	0,63 [0,09 - 15,26]	0,444
FABP4 (pg/mL)	0,21 [0,06 - 0,67]	0,86 [0,14 - 1,32]	0,82 [0,10 - 17,05]	0,248
LIGHT (pg/mL)	0,06 [0,02 - 1,21]	0,86 [0,06 - 46,41]	0,42 [0,12 - 0,92]	0,203
GDF-15 (ng/mL)	11,38 [3,82 - 15,15]	14,75 [11,10 - 19,54]	24,79 [17,92 - 1899,90]	0,030 ^†^
sCD14 (pg/mL)	3,387 [0,026 - 5,574]	4,098 [1,362 - 5,031]	0,021 [0,005 - 4,767]	0,398
ucMGP (ng/mL)	0,09 [0,05 - -18,93]	0,15 [0,09 - 0,29]	7,97 [0,06 - 15,87]	0,957

CXCL-16: Ligante do motivo C-X-C 16; FABP3: proteína de ligação a ácidos graxos 3; FABP4: proteína de ligação a ácidos graxos 4 LIGHT: membro da superfamília do fator de necrose tumoral 14/TNFS14; GDF-15: Fator de crescimento/diferenciação 15; sCD4: forma solúvel de CD14; ucMGP: Proteína Gla de matriz não carboxilada. ^†^ Não significativo após correção de Bonferroni.

**Tabela 3:** – Biomarcadores cardiovasculares segundo risco cardiovascular de Framingham em pacientes com câncer de mama

Biomarcadores	Baixo risco (n=15)	Risco intermediário (n=10)	Alto risco (n=9)	Valor de p
CXCL-16 (pg/mL)	1,34 [1,19 – 1,90]	1,40 [0,96 – 1,72]	2,32 [1,43 – 2,83]	0,101
FABP3 (pg/mL)	1,00 [0,59 – 1,27]	1,37 [1,14 – 12,21]	11,37 [1,06 – 19,54]	0,022 ^†^
FABP4 (pg/mL)	0,15 [0,08 – 0,62]	1,18 [0,48 – 1,41]	0,84 [0,08 – 1,41]	0,096
LIGHT (pg/mL)	0,41 [0,06 – 35,08]	0,06 [0,02 – 0,55]	4,35 [0,16 – 25,33]	0,115
GDF-15 (ng/mL)	11,74 [1,72 – 15,66]	14,91 [11,82 – 19,75]	17,53 [11,82 – 24,02]	0,066
sCD14 (pg/mL)	5,18 [3,51 – 6,78]	0,28 [0,02 – 5,23]	2,24 [0,47 – 4,52]	0,080
ucMGP (ng/mL)	0,16 [0,09 – 14,07]	0,88 [0,05 – 14,05]	0,11 [0,06 – 0,36]	0,620

CXCL-16: Ligante do motivo C-X-C 16; FABP3: proteína de ligação a ácidos graxos 3; FABP4: proteína de ligação a ácidos graxos 4 LIGHT: membro da superfamília do fator de necrose tumoral 14/TNFS14; GDF-15: Fator de crescimento/diferenciação 15; sCD4: forma solúvel de CD14; ucMGP: Proteína Gla de matriz não carboxilada. ^†^ Não significativo após correção de Bonferroni.

## Discussão

A investigação, monitoramento e avaliação de lesões cardiovasculares em pacientes com câncer de mama em regime de quimioterapia têm sido amplamente estudadas. No entanto, estudos incluindo biomarcadores emergentes capazes de detectar precocemente o comprometimento cardiovascular em pacientes com câncer de mama sob quimioterapia baseada em DOXO são raros, independente da cardiotoxicidade clínica. Assim, as principais conclusões do presente estudo são: (i) pacientes com câncer de mama apresentaram níveis mais elevados de GDF-15, mostrando boa precisão para diferenciar esse grupo e os controles, de acordo com a área sob a curva de ROC; (ii) pacientes com câncer de mama apresentaram níveis mais baixos de FABP3, FABP4, sCD14 e ucMGP; e (iii) houve correlação positiva entre FABPs e IMC.

O GDF-15 é um preditor forte e independente de DCV, morbidade e mortalidade por câncer em indivíduos residentes em comunidades. ^30^ O grupo com câncer de mama apresentou níveis de GDF-15 8,12 vezes maiores do que os indivíduos saudáveis. No câncer de mama, o GDF-15 tem sido associado a metástases e resistência ao trastuzumabe. ^31^ O GDF-15 aumenta devido a diversas condições fisiopatológicas; portanto, níveis elevados de GDF-15 devem ser interpretados com cautela. Neste estudo, as razões associadas ao aumento dos níveis de GDF-15 em pacientes com câncer de mama permanecem obscuras, uma vez que a biologia do câncer de mama e a quimioterapia baseada em DOXO são ambas condições que podem promover alterações no GDF-15. O GDF-15 é um biomarcador emergente que se encontra elevado na doença subclínica inicial e tem utilidade prognóstica para eventos cardiovasculares e mortalidade. ^32^ Assim, estudos mais robustos de caso-controle, que incluam pelo menos um grupo de pacientes com câncer de mama tratados com outra classe de medicamentos quimioterápicos, poderiam ser benéficos para esclarecer a hipótese desse estudo.

Uma coorte prospectiva de Demissei et al. ^6^ incluiu 323 pacientes com câncer de mama tratados com regimes baseados em antraciclina e/ou trastuzumabe. Nesse estudo, nenhuma associação entre os níveis de GDF-15, troponina, mieloperoxidase e fator de crescimento placentário com alterações na FEVE foram encontrados. Além disso, não foram observadas alterações nos níveis de GDF-15 nos dois anos de estudo. Na linha de base, os níveis de GDF-15 em pacientes com câncer de mama, que receberam tratamento com DOXO, foram de 704 [532–908] pg/mL e 599 [523–722] pg/mL para pacientes que receberam DOXO+Trastuzumabe. No presente estudo, o GDF-15 foi maior nos pacientes triplo negativos, mas a diferença não foi significativa, requerendo mais estudos com uma maior população. Em um estudo de coorte multicêntrico, os níveis de GDF-15 permaneceram elevados mesmo após 15 meses de estudo em pacientes com câncer da mama (HER2+) sob terapêutica adjuvante com um regime contendo antraciclina seguido de taxanos e trastuzumabe. ^33^

Os níveis de FABP4 foram mais baixos em pacientes com câncer de mama em comparação com o grupo controle do presente estudo, sendo um achado inesperado de acordo com Tsakogiannis et al. ^34^ A FABP4 é altamente expressa em adipócitos, mas pacientes com câncer de mama não apresentaram diferenças no IMC em comparação com controles saudáveis. Porém, o IMC não é o melhor método para avaliar a adiposidade; outros marcadores da composição da gordura corporal devem ser aplicados para correlacionar com os níveis de FABP4. Na verdade, seus níveis mostraram correlação com o IMC no grupo com câncer de mama no presente estudo. Contrariamente às observações aqui registradas, outro estudo de caso-controle encontrou níveis mais elevados de FABP4 em pacientes com câncer de mama, em contraste com mulheres saudáveis, e níveis mais elevados no câncer de mama do tipo luminal em comparação com HER2+/triplo negativo. No entanto, eles também sugerem que o IMC no câncer de mama pode ser um fator que afeta a expressão de FABP4, já que pacientes com câncer de mama e IMC ≥25 kg/m ^2^ apresentaram níveis mais elevados de FABP4. ^34^ Estes dados enfatizam que as FABPs são expressas pelo tecido adiposo. Além disso, a FABP4 circulante aumenta o fenótipo semelhante a células-tronco tumorais por meio da atividade mediada pelo IL-6/STAT3/ALDH1, sugerindo que a FABP4 circulante liberada pelo tecido adiposo do hospedeiro pode desencadear a saída da dormência tumoral ^35^ e que a FABP-4 é regulada positivamente em determinados subconjuntos de macrófagos em tumores de mama, o que aumenta sua capacidade de promover o crescimento tumoral e a metástase por meio das vias dependentes de IL-6. ^36^

Os níveis de FABP3 também foram mais baixos no grupo com câncer de mama em comparação com o grupo controle. Sabe-se que a FABP3 desempenha um papel fundamental no metabolismo dos cardiomiócitos. No entanto, é possível levantar a hipótese de que a quimioterapia com DOXO poderia promover uma diminuição na síntese de FABP3 no tecido cardíaco, uma vez que a DOXO induz a apoptose de cardiomiócitos. ^37^ Isto foi demonstrado por Sayed-Ahmed et al., ^38^ cujos experimentos mostram que o uso crônico de DOXO resultou em diminuição significativa e dose-dependente na expressão de mRNA de FABP3 no tecido cardíaco. Além disso, a perda da homeostase do metabolismo lipídico celular devido à redução do conteúdo intracelular de FABP3 e ao fornecimento prejudicado de ácidos graxos parece ser uma hipótese plausível para a progressão da insuficiência cardíaca e de outras DCV. ^39^

Conway et al. demonstraram que o promotor da FABP3 estava hipermetilado e a expressão gênica estava reduzida no câncer de mama, indicando que a expressão de FABP3 tem um efeito inibitório na doença. ^40^ Por outro lado, a FABP3 é um marcador de lesão cardíaca, uma vez que níveis elevados são úteis para o diagnóstico precoce do infarto agudo do miocárdio. ^41^ Os níveis plasmáticos de FABP3 já foram investigados no contexto da quimioterapia do câncer de mama. No entanto, não foram observadas diferenças em indivíduos que desenvolveram cardiotoxicidade secundária às antraciclinas em comparação com indivíduos sem cardiotoxicidade. ^42^ Estudos experimentais e clínicos substanciais são necessários para esclarecer o comportamento da FABP3 neste contexto. A correlação positiva entre FABP3 e FABP4 com o IMC já era esperada, uma vez que esses marcadores estão diretamente associados ao tecido adiposo e ao metabolismo lipídico.

Embora níveis mais baixos de sCD14 tenham sido observados em pacientes com câncer de mama no presente estudo, esse achado é controverso, uma vez que alguns estudos mostraram que os níveis de sCD14 estavam mais elevados em pacientes com câncer do que em pacientes com doença benigna ou em indivíduos saudáveis. ^43 , 44^ O resultado do CD14 na inflamação é multifatorial, incluindo o local da inflamação, o nível de expressão do CD14, as características dos ligantes do CD14 e a competição entre diferentes vias dependentes do CD14. ^21^ O CD14 também é expresso nas membranas celulares dos cardiomiócitos ^45^ e o efeito apoptótico da DOXO nos cardiomiócitos pode reduzir o CD14 solúvel. Além disso, os níveis de sCD14 também foram determinados em outros estudos como reagente de fase aguda, ^46^ mas os pacientes avaliados não estavam em fase inflamatória aguda, conforme avaliada por medidas da proteína C reativa (PCR) (dados não mostrados). Assim, estes resultados devem ser interpretados com cautela e estudos futuros neste contexto devem ser realizados a fim de determinar o papel do sCD14 neste cenário.

Pacientes com câncer de mama também apresentaram níveis plasmáticos de ucMGP mais baixos em comparação com indivíduos controle. De acordo com Yoshimura et al., ^47^ o gene MGP é regulado positivamente em casos de prognóstico ruim, indicando que os níveis de mRNA do MGP são um potencial indicador prognóstico do câncer de mama. No entanto, não houve diferença na expressão proteica do tumor por meio de imuno-histoquímica. Por outro lado, níveis mais baixos de ucMGP foram causalmente relacionados a uma diminuição do risco de doença coronariana. ^48^ As formas inativas de MGP (como uc-MGP) são biomarcadores úteis de deficiência de vitamina K, calcificação vascular e DCV e podem prever um risco futuro de morte ou eventos cardiovasculares. Trata-se de uma proteína dependente de vitamina K (VKDP), liberada a partir das células na corrente sanguínea. A vitamina K atenua as respostas inflamatórias bloqueando a transdução do sinal do fator nuclear κB (NF-κB). Níveis mais elevados de ucMGP refletem a calcificação vascular, que é um dos principais fatores de risco de morbidade e mortalidade cardiovascular. ^49 , 50^ Desta forma, os dados do presente estudo sugerem que a administração de DOXO não induz calcificação cardiovascular a curto prazo, um mecanismo improvável relacionado à toxicidade cardiovascular da DOXO. A determinação dos níveis de vitamina K, como citocinas pró-inflamatórias (como IL-6 e TNF-α; que aceleram a formação de VKDPs) e a quantificação de outras VKDPs, como osteocalcina e 6 específico para parada de crescimento (Gas6) e proteína rica em Gla (GRP) é fortemente encorajada em futuros estudos clínicos prospectivos, incluindo pacientes com câncer de mama sob tratamento com DOXO.

### Limitações

Este estudo apresenta limitações, como um estudo seccional de centro único, realizado com pacientes que faziam quimioterapia apenas com DOXO. O tamanho pequeno da amostra também é uma limitação importante do presente estudo. Além disso, como os biomarcadores não foram avaliados antes do tratamento, o próprio câncer poderia influenciar alguns resultados. Consequentemente, novos estudos longitudinais para validação desses marcadores, com uma população maior, devem ser realizados para avaliar seu desempenho no monitoramento das alterações cardiovasculares causadas pela quimioterapia baseada em DOXO.

## Conclusão

O resultado deste estudo é preliminar, mas pode contribuir para uma melhor compreensão dos mecanismos subjacentes à cardiotoxicidade secundária à quimioterapia baseada em DOXO. Além disso, os resultados sugerem que os níveis de GDF-15, FABP3, FABP4, sCD14 e ucMGP podem estar relacionados a alterações cardiovasculares em pacientes com câncer de mama tratados com DOXO. Mais estudos devem ser realizados em outras populações para validar os presentes resultados.

## References

[1] Mansur AP, Favarato D (2021). Cardiovascular and Cancer Death Rates in the Brazilian Population Aged 35 to 74 Years, 1996-2017. Arq Bras Cardiol.

[2] Mehta LS, Watson KE, Barac A, Beckie TM, Bittner V, Cruz-Flores S (2018). Cardiovascular Disease and Breast Cancer: Where These Entities Intersect: A Scientific Statement from the American Heart Association. Circulation.

[3] Maki Y, Sueta D, Ishii M, Yamanouchi Y, Fujisue K, Yamanaga K (2021). Associations of Cardiovascular Risk Factors with Survival Outcomes in a Cancer Registration: Findings from the KUMAMON Registry. Medicine.

[4] Leiva O, AbdelHameid D, Connors JM, Cannon CP, Bhatt DL (2021). Common Pathophysiology in Cancer, Atrial Fibrillation, Atherosclerosis, and Thrombosis: JACC: CardioOncology State-of-the-Art Review. JACC CardioOncol.

[5] Peto R, Davies C, Godwin J, Gray R, Pan HC, Early Breast Cancer Trialists’ Collaborative Group (EBCTCG) (2012). Comparisons between Different Polychemotherapy Regimens for Early Breast Cancer: Meta-Analyses of Long-Term Outcome Among 100,000 Women in 123 Randomised Trials. Lancet.

[6] Demissei BG, Hubbard RA, Zhang L, Smith AM, Sheline K, McDonald C (2020). Changes in Cardiovascular Biomarkers with Breast Cancer Therapy and Associations with Cardiac Dysfunction. J Am Heart Assoc.

[7] Rüger AM, Schneeweiss A, Seiler S, Tesch H, van Mackelenbergh M, Marmé F (2020). Cardiotoxicity and Cardiovascular Biomarkers in Patients with Breast Cancer: Data from the GeparOcto-GBG 84 Trial. J Am Heart Assoc.

[8] Pestana RMC, Duarte RCF, Alves MT, de Oliveira AN, Oliveira HHM, Soares CE (2022). Hemostatic Status in Women with Breast Cancer and Cardiotoxicity Associated to Doxorubicin-Based Chemotherapy - A One-Year Follow-Up Study. Thromb Res.

[9] Herrmann J, Lenihan D, Armenian S, Barac A, Blaes A, Cardinale D (2022). Defining Cardiovascular Toxicities of Cancer Therapies: An International Cardio-Oncology Society (IC-OS) Consensus Statement. Eur Heart J.

[10] López-Sendón J, Álvarez-Ortega C, Zamora Auñon P, Buño Soto A, Lyon AR, Farmakis D (2020). Classification, Prevalence, and Outcomes of Anticancer Therapy-Induced Cardiotoxicity: The CARDIOTOX Registry. Eur Heart J.

[11] Pradhan NM, Mullin C, Poor HD (2020). Biomarkers and Right Ventricular Dysfunction. Crit Care Clin.

[12] Cardinale D, Colombo A, Bacchiani G, Tedeschi I, Meroni CA, Veglia F (2015). Early Detection of Anthracycline Cardiotoxicity and Improvement with Heart Failure Therapy. Circulation.

[13] Sjöberg E, Meyrath M, Chevigné A, Östman A, Augsten M, Szpakowska M (2020). The Diverse and Complex Roles of Atypical Chemokine Receptors in Cancer: From Molecular Biology to Clinical Relevance and Therapy. Adv Cancer Res.

[14] Korbecki J, Bajdak-Rusinek K, Kupnicka P, Kapczuk P, Simińska D, Chlubek D (2021). The Role of CXCL16 in the Pathogenesis of Cancer and Other Diseases. Int J Mol Sci.

[15] Wang J, Lo JC, Foster A, Yu P, Chen HM, Wang Y (2001). The Regulation of T Cell Homeostasis and Autoimmunity by T Cell-Derived LIGHT. J Clin Invest.

[16] Skeate JG, Otsmaa ME, Prins R, Fernandez DJ, Da Silva DM, Kast WM (2020). TNFSF14: LIGHTing the Way for Effective Cancer Immunotherapy. Front Immunol.

[17] Wischhusen J, Melero I, Fridman WH (2020). Growth/Differentiation Factor-15 (GDF-15): From Biomarker to Novel Targetable Immune Checkpoint. Front Immunol.

[18] Bledsoe G, Crickman S, Mao J, Xia CF, Murakami H, Chao L (2006). Kallikrein/Kinin Protects Against Gentamicin-Induced Nephrotoxicity by Inhibition of Inflammation and Apoptosis. Nephrol Dial Transplant.

[19] Cranenburg EC, Vermeer C, Koos R, Boumans ML, Hackeng TM, Bouwman FG (2008). The Circulating Inactive Form of Matrix Gla Protein (ucMGP) as a Biomarker for Cardiovascular Calcification. J Vasc Res.

[20] Mayer O, Seidlerová J, Bruthans J, Filipovský J, Timoracká K, Vaněk J (2014). Desphospho-Uncarboxylated Matrix Gla-Protein is Associated with Mortality Risk in Patients with Chronic Stable Vascular Disease. Atherosclerosis.

[21] Wu Z, Zhang Z, Lei Z, Lei P (2019). CD14: Biology and Role in the Pathogenesis of Disease. Cytokine Growth Factor Rev.

[22] Wright SD, Ramos RA, Tobias PS, Ulevitch RJ, Mathison JC (1990). CD14, a Receptor for Complexes of Lipopolysaccharide (LPS) and LPS Binding Protein. Science.

[23] Thumser AE, Moore JB, Plant NJ (2014). Fatty Acid Binding Proteins: Tissue-Specific Functions in Health and Disease. Curr Opin Clin Nutr Metab Care.

[24] Zhen EY, Berna MJ, Jin Z, Pritt ML, Watson DE, Ackermann BL (2007). Quantification of Heart Fatty Acid Binding Protein as a Biomarker for Drug-Induced Cardiac and Musculoskeletal Necroses. Proteomics Clin Appl.

[25] Mion MM, Novello E, Altinier S, Rocco S, Zaninotto M, Plebani M (2007). Analytical and Clinical Performance of a Fully Automated Cardiac Multi-Markers Strategy Based on Protein Biochip Microarray Technology. Clin Biochem.

[26] Niizeki T, Takeishi Y, Arimoto T, Takabatake N, Nozaki N, Hirono O (2007). Heart-Type Fatty Acid-Binding Protein is More Sensitive than Troponin T to Detect the Ongoing Myocardial Damage in Chronic Heart Failure Patients. J Card Fail.

[27] Furuhashi M (2019). Fatty Acid-Binding Protein 4 in Cardiovascular and Metabolic Diseases. J Atheroscler Thromb.

[28] D‘Agostino RB, Vasan RS, Pencina MJ, Wolf PA, Cobain M, Massaro JM (2008). General Cardiovascular Risk Profile for use in Primary Care: The Framingham Heart Study. Circulation.

[29] Simões R, Silva LM, de Oliveira AN, Alves MT, Pestana RMC, de Souza IDP (2021). Identification of Clinical and Laboratory Variables Associated with Cardiotoxicity Events Due to Doxorubicin in Breast Cancer Patients: A 1-Year Follow-Up Study. Cardiovasc Toxicol.

[30] Wollert KC, Kempf T, Wallentin L (2017). Growth Differentiation Factor 15 as a Biomarker in Cardiovascular Disease. Clin Chem.

[31] Blanchette-Farra N, Kita D, Konstorum A, Tesfay L, Lemler D, Hegde P (2018). Contribution of Three-Dimensional Architecture and Tumor-Associated Fibroblasts to Hepcidin Regulation in Breast Cancer. Oncogene.

[32] Schopfer DW, Ku IA, Regan M, Whooley MA (2014). Growth Differentiation Factor 15 and Cardiovascular Events in Patients with Stable Ischemic Heart Disease (The Heart and Soul Study). Am Heart J.

[33] Putt M, Hahn VS, Januzzi JL, Sawaya H, Sebag IA, Plana JC (2015). Longitudinal Changes in Multiple Biomarkers are Associated with Cardiotoxicity in Breast Cancer Patients Treated with Doxorubicin, Taxanes, and Trastuzumab. Clin Chem.

[34] Tsakogiannis D, Kalogera E, Zagouri F, Zografos E, Balalis D, Bletsa G (2021). Determination of FABP4, RBP4 and the MMP-9/NGAL Complex in the Serum of Women with Breast Cancer. Oncol Lett.

[35] Prentice KJ, Saksi J, Hotamisligil GS (2019). Adipokine FABP4 Integrates Energy Stores and Counterregulatory Metabolic Responses. J Lipid Res.

[36] Hao J, Yan F, Zhang Y, Triplett A, Zhang Y, Schultz DA (2018). Expression of Adipocyte/Macrophage Fatty Acid-Binding Protein in Tumor-Associated Macrophages Promotes Breast Cancer Progression. Cancer Res.

[37] Rawat PS, Jaiswal A, Khurana A, Bhatti JS, Navik U (2021). Doxorubicin-Induced Cardiotoxicity: An Update on the Molecular Mechanism and Novel Therapeutic Strategies for Effective Management. Biomed Pharmacother.

[38] Sayed-Ahmed MM, Al-Shabanah OA, Hafez MM, Aleisa AM, Al-Rejaie SS (2010). Inhibition of Gene Expression of Heart Fatty Acid Binding Protein and Organic Cation/Carnitine Transporter in Doxorubicin Cardiomyopathic Rat Model. Eur J Pharmacol.

[39] Rezar R, Jirak P, Gschwandtner M, Derler R, Felder TK, Haslinger M (2020). Heart-Type Fatty Acid-Binding Protein (H-FABP) and its Role as a Biomarker in Heart Failure: What do we Know so Far?. J Clin Med.

[40] Conway K, Edmiston SN, May R, Kuan PF, Chu H, Bryant C (2014). DNA Methylation Profiling in the Carolina Breast Cancer Study Defines Cancer Subclasses Differing in Clinicopathologic Characteristics and Survival. Breast Cancer Res.

[41] Lippi G, Mattiuzzi C, Cervellin G (2013). Critical Review and Meta-Analysis on the Combination of Heart-Type Fatty Acid Binding Protein (H-FABP) and Troponin for Early Diagnosis of Acute Myocardial Infarction. Clin Biochem.

[42] Caballero RM, Antolin JMS, Garcia IAG, Garcia JMM, Ruigomez AC, Landaluce CG (2019). Incidence and Predictors of Long Term Cardiotoxicity in Antracycline Based Chemotherapy in Breast Cancer Patients. J Am Coll Cardiol.

[43] Wu CC, Hsu CW, Chen CD, Yu CJ, Chang KP, Tai DI (2010). Candidate Serological Biomarkers for Cancer Identified from the Secretomes of 23 Cancer Cell Lines and the Human Protein Atlas. Mol Cell Proteomics.

[44] Gadducci A, Ferdeghini M, Castellani C, Annicchiarico C, Gagetti O, Prontera C (1995). Serum Levels of Tumor Necrosis Factor (TNF), Soluble Receptors for TNF (55- and 75-kDa sTNFr), and Soluble CD14 (sCD14) in Epithelial Ovarian Cancer. Gynecol Oncol.

[45] Cowan DB, Poutias DN, Del Nido PJ, McGowan FX (2000). CD14-Independent Activation of Cardiomyocyte Signal Transduction by Bacterial Endotoxin. Am J Physiol Heart Circ Physiol.

[46] Bas S, Gauthier BR, Spenato U, Stingelin S, Gabay C (2004). CD14 is an Acute-Phase Protein. J Immunol.

[47] Yoshimura K, Takeuchi K, Nagasaki K, Ogishima S, Tanaka H, Iwase T (2009). Prognostic Value of Matrix Gla Protein in Breast Cancer. Mol Med Rep.

[48] Zwakenberg SR, Burgess S, Sluijs I, Weiderpass E, Beulens JWJ, EPIC-CVD consortium (2020). Circulating Phylloquinone, Inactive Matrix Gla Protein and Coronary Heart Disease Risk: A Two-Sample Mendelian Randomization Study. Clin Nutr.

[49] Shioi A, Morioka T, Shoji T, Emoto M (2020). The Inhibitory Roles of Vitamin K in Progression of Vascular Calcification. Nutrients.

[50] Rapp N, Brandenburg VM, Kaesler N, Bakker SJL, Stöhr R, Schuh A (2021). Hepatic and Vascular Vitamin K Status in Patients with High Cardiovascular Risk. Nutrients.

