# Supplementary material for: Novos Biomarcadores Cardiovasculares em Pacientes com Câncer de Mama Submetidas a Quimioterapia à Base de Doxorrubicina
Source: Arq Bras Cardiol. 2023 Dec 14;120(12):e20230167. [Article in Portuguese] doi: 10.36660/abc.20230167 (PMC10789374; doi:10.36660/abc.20230167)
Supplement: Translation

# Translation: New Cardiovascular Biomarkers in Breast Cancer Patients Undergoing Doxorubicin-Based Chemotherapy

**Authors:** Rodrigo Mendonça Cardoso Pestana, Júnea Paolucci Paiva Silvino, Angélica Navarro de Oliveira, Cintia Esteves Soares, Adriano de Paula Sabino, Ricardo Simões, Karina Braga Gomes

**Affiliations:** 1 Brazil Faculdade de Medicina – Universidade Federal de Minas Gerais, Belo Horizonte, MG – Brazil; 2 Brazil Instituto de Hipertensão, Belo Horizonte, MG – Brazil; 3 Brazil Fundação Hospitalar do Estado de Minas Gerais (FHEMIG), Belo Horizonte, MG – Brazil; 4 Brazil Faculdade de Farmácia - Universidade Federal de Minas Gerais, Belo Horizonte, MG – Brazil

**Keywords:** Breast Neoplasms, Cardiovascular Diseases, Doxorubicin, Biomarkers

## Abstract

**Background:**

Cardiovascular diseases (CVDs) are relevant to the management of breast cancer treatment since a substantial number of patients develop these complications after chemotherapy.

**Objective:**

This study aims to evaluate new cardiovascular biomarkers, namely CXCL-16 (C-X-C motif ligand 16), FABP3 (fatty acid binding protein 3), FABP4 (fatty acid binding protein 4), LIGHT (tumor necrosis factor superfamily member 14/TNFS14), GDF-15 (Growth/differentiation factor 15), sCD4 (soluble form of CD14), and ucMGP (uncarboxylated Matrix Gla-Protein) in breast cancer patients treated with doxorubicin (DOXO).

**Methods:**

This case-control study was conducted in an oncology clinic that included 34 women diagnosed with breast cancer and chemotherapy with DOXO and 34 control women without cancer and CVD. The markers were determined immediately after the last cycle of chemotherapy. The statistical significance level adopted was 5%.

**Results:**

The breast cancer group presented higher levels of GDF-15 (p<0.001), while control subjects had higher levels of FABP3 (p=0.038), FABP4 (p=0003), sCD14, and ucMGP (p<0.001 for both). Positive correlations were observed between FABPs and BMI in the cancer group.

**Conclusion:**

GDF15 is an emerging biomarker with potential clinical applicability in this scenario. FABPs are proteins related to adiposity, which are potentially involved in breast cancer biology. sCD14 and ucMGP engage in inflammatory and vascular calcification. The evaluation of these novel cardiovascular biomarkers could be useful in the management of breast cancer chemotherapy with DOXO.

## Introduction

Cardiovascular diseases (CVDs) and cancer are the leading causes of death worldwide. ^1^ Continuous improvements in strategies for prevention and anti-cancer treatments in patients with breast cancer significantly reduced the death from cancer-related causes; however, there was an increased risk of death from CVD in this group of patients. ^2^ The reasons for this synergism between cancer and CVD are the common risk factors (including diabetes mellitus, hypertension, hypercholesterolemia, and obesity), as well as pathophysiological mechanisms underlying CVD that are associated with an increased risk of cancers. ^3 , 4^

Anthracycline-based regimens, like doxorubicin (DOXO), are some of the most effective treatments against breast cancer and are responsible for improved disease-free survival and overall survival in this group. Nevertheless, anthracyclines can result in severe short and long-term toxicity, including cardiotoxicity and secondary hematological malignancy. ^5^

Many studies have proposed the use of plasma biomarkers, especially troponins and B-type natriuretic peptides, to monitor anthracycline cardiotoxicity for the early detection of these cardiovascular complications. ^6 - 8^ More recently, these biomarkers have been included as diagnosis criteria of cardiotoxicity in addition to cardiologic imaging exams and their modalities and clinical features. ^9 , 10^ Other biomarkers underlie the pathophysiological changes that occur during heart failure. Heart failure manifests as decreased left ventricular ejection fraction (LVEF) or symptomatic heart failure in up to 5% of patients. ^11^ In a prospective study, Cardinale et al. ^12^ found an overall incidence of cardiotoxicity of 9% using the decrease in LVEF as a single criterion to define cardiotoxicity. However, in another prospective study, López-Séndon et al. ^10^ expanded the criteria for defining cardiotoxicity beyond changes in LVEF, including the use of plasma biomarkers and cardiotoxicity, which was identified in 37.5% of patients during the follow-up.

Chemokines are pro-inflammatory chemoattractant cytokines that act primarily in leukocyte trafficking, regulate cell migration, proliferation, and survival, being key components in cancer biology. ^13^ CXCL-16 (C-X-C motif ligand 16) is a chemokine expressed in lymphoid organs, the liver, lungs, small intestine, and kidney. CXCL-16 expression is increased by pro-inflammatory cytokines, important for the accumulation of immune cells at the inflammatory reaction sites. ^14^

LIGHT (tumor necrosis factor superfamily member 14/TNFS14) belongs to the tumor necrosis factor superfamily and is expressed by numerous types of immune cells. LIGHT signals through two receptors and has distinct functions that are cell-type dependent, but interactions with these types of receptors have immune-related implications in tumor biology. ^15 , 16^

Growth/differentiation factor 15 (GDF-15) is a divergent member of the transforming growth factor-β (TGF-β) superfamily and is also known as macrophage inhibitory cytokine (MIC)-1. ^17^ GDF-15 is also related to the evolution of cancer both positively and negatively, since GDF-15 inhibits early tumor promotion, but its abnormal expression in advanced cancers causes cancer stem cell formation, proliferation, invasion, metastasis, immune escape, and a reduced response to therapy. ^18^

Matrix-carboxyglutamate (Gla) protein (MGP) is a vitamin K-dependent protein and a strong inhibitor of vascular calcification. Vitamin K deficiency leads to inactive uncarboxylated MGP (ucMGP), which accumulates at sites of arterial calcification. ^19^ Desphospho-ucMGP is a biologically inactive marker of vascular vitamin K status and is described to predict mortality in patients with heart failure and aortic stenosis. ^20^

The human monocyte differentiation antigen CD14 is a pattern recognition receptor (PRR) that enhances innate immune responses. CD14 was first identified as a marker of monocytes to signal intracellular responses upon bacterial encounters. ^21^ Soluble forms of CD14 (sCD14) could be secreted by activated cells, which release CD14 by proteinase-dependent or -independent shedding. ^22^

The FABPs (fatty acid binding proteins) are expressed in proteins in almost all tissues. These proteins are responsible for the control of fat acid transport, metabolism, and storage. FABPs are proposed to be central regulators of lipid metabolism, inflammation, and energy homeostasis. ^23^ FABP3 is a cytosolic protein found primarily in the heart but also in the muscle, brain, and kidney. ^24^ Some studies have suggested that FABP3 has superior sensitivity to troponin for detecting ischemic injury and cardiac injury associated with congestive heart failure. ^25 , 26^ FABP4 is mainly expressed in adipocytes and macrophages and plays a significant role in the development of insulin resistance and atherosclerosis. Circulating FABP4 levels are associated with several aspects of metabolic syndrome and cardiovascular disease. ^27^

The implementation of new laboratory biomarkers has been a priority in cardio-oncology, particularly for early detection of cardiotoxicity secondary to chemotherapy. In this context, this study aimed to evaluate new cardiovascular biomarkers, such as CXCL-16, FABP4, LIGHT, GDF-15, sCD14, and ucMGP in breast cancer patients under DOXO-based chemotherapy.

## Subjects and Methods

### Human samples

This is a case-control study performed with outpatients from the Oncology Service at Alberto Cavalcanti Hospital/FHEMIG (Belo Horizonte, Brazil), which included 34 women aged 18 or older diagnosed with breast cancer and using neoadjuvant therapy with DOXO, who were attended from June 2015 to June 2018. The exclusion criteria for the case group were: presence of previous heart disease with impaired left ventricular function; moderate to severe hepatic or renal dysfunction; degenerative brain diseases requiring caregivers; and pregnant women or patients with a life expectancy shorter than three months. Moreover, women submitted to previous chemotherapy, hormone therapy, immunotherapy, or radiation therapy were excluded. The control group was composed of 34 healthy subjects aged 18 or older without any malignant disease or the presence of previous heart disease, moderate to severe hepatic or renal dysfunction, degenerative diseases, and no pregnancy, attested by a clinical physician.

Clinical characteristics of breast cancer patients were obtained from hospital medical records. Prior to chemotherapy, patients with breast cancer underwent a medical evaluation with a certified cardiologist, who performed an electrocardiogram and a two-dimensional echocardiogram, including tissue mode with Vivid S6 echocardiographic (GE Medical Systems Healthcare ^®^ , Tirat Carmel, Israel) with LVEF assessment. No alterations in these exams were observed. The cardiovascular risk of patients with breast cancer was calculated according to the global risk score (Framingham Heart Study) ^28^ before cancer treatment.

The study was approved by the UFMG Research Ethics Committee (n. 38538714.20000.5149) and FHEMIG Ethics Committee (n. 54376216.0.0000.5119), pursuant to the World Medical Association Declaration of Helsinki. All participants signed the informed consent form beforehand.

### Experimental and laboratory protocols

Fasting blood collection was conducted after the DOXO-based chemotherapy (out up to seven days after the last DOXO cycle). For plasma preparation, the EDTA tube was centrifuged for 10 minutes at 1000 g within 30 minutes of blood collection and, for serum preparation, the tube without additive was centrifuged at 3000 g for 15 min. The plasma and serum samples were distributed in aliquots and immediately stored at -80ºC until assayed.

The cardiovascular markers’ levels were determined by Multiplexed Immunoassays using a Luminex ^®^ xMAP ^®^ platform. EDTA-plasma was used for determinations of CXCL-16, FABP3, FABP4, LIGHT (HCVD1MAG-67K kit; Merck ^®^ , Darmstadt, Germany), GDF-15 (HCVD2MAG-67K kit; Merck ^®^ , Darmstadt, Germany), sCD14, and ucMGP (HCVD6MAG-67K kit; Merck ^®^ , Darmstadt, Germany), according to manufacturer’s instructions in a MAGPIX ^®^ Multiplexing System Analyzer (Luminex Corporation ^®^ , Austin, USA).

Levels of cTnI (troponine I) and NT-proBNP (fraction NT of natriuretic peptide type B), as well as LVEF to monitor cardiac dysfunction assessment, were determined according to the protocols described in a previous study. ^29^ Total cholesterol and HDL-cholesterol were performed by colorimetric assay on VITROS 5600 (Ortho Clinical Diagnostics ^®^ , Rochester, USA). LDL-cholesterol was calculated by Friedwald formula.

### Statistical analysis

Data were analyzed using IBM ^®^ SPSS ^®^ Statistics software (for Windows ^®^ ; Chicago, Illinois, USA, version 21). The Shapiro-Wilk test was used to verify the normality of quantitative variables, which were presented as the mean ± standard deviation (SD) or median (25 ^th^ –75 ^th^ percentiles). Unpaired Student’s t test and One-Way ANOVA (followed by the Tukey test) or the Mann-Whitney and Kruskal-Wallis test (followed by the Bonferroni test) determined the differences between two and three groups, as appropriate. Categorical variables were presented as n (%) and compared by Fisher’s exact test. Correlations were performed using Spearman’s correlation test. Receiver operating characteristic (ROC) curves were used to represent the sensitivity and specificity. The significance level adopted was 5%.

## Results

Mean age and body mass index (BMI) of patients with breast cancer prior to chemotherapy and controls are presented in Table 1 , and no difference between the groups was observed. Regarding arterial hypertension and diabetes mellitus, the breast cancer group presented higher frequencies than controls (all p<0.05). After the treatment, all breast cancer patients’ ventricular function was normal (LVEF ≥50%). Eleven (32.3%) patients presented NT-proBNP levels above the reference value (<125.0 pg/mL if <75 years, or <450.0 pg/mL, if ≥75 years), but no alteration on cTnI levels (normal range <0.120 ng/mL) was found in the breast cancer group. No patient presented clinical cardiotoxicity. Other characteristics of breast cancer patients are summarized in Table 1 .

**Table 1:** – Breast cancer patients’ clinical characteristics prior to DOXO-chemotherapy and controls

Variable	Breast Cancer (n=34)	Controls (n=34)	p-value
**Age (years)**	50.2 ± 11.3	46.9 ± 16.9	0.349
**BMI (kg/m** ^**2**^ **)**	27.91 ± 5.9	26.5 ± 6.2	0.340
**Diabetes mellitus, yes (n, %)**	3 (8.8)	2 (5.8)	<0.001*
**Arterial hypertension, yes (n, %)**	11 (32.4)	9 (26.8)	0.008*
**DOXO dose (mg/m** ^**2**^ **)**	380.6 [360.0 – 400.0]	-	-
**LVEF (%)**	66.97 ± 2.33	-	-
**Histological diagnosis, n (%)**			
Invasive ductal carcinoma	30 (88.2)		
Lobular carcinoma	3 (8.8)		
Special types	1 (2.9)	-	-
**Molecular type**			
HER2+	18 (52.9)		
Luminal	12 (35.3)		
Triple negative	4 (11.8)	-	-

BMI: body mass index; DOXO: doxorubicin; LVEF: left ventricular ejection fraction. * Significant p-value <0.050.

The comparison of cardiovascular markers between breast cancer and the control group is described in Figure 1 . Breast cancer patients had higher plasma levels of GDF-15 (p<0.001) and lower FABP3 (p=0.038), FABP4 (p=0.003), sCD14, and ucMGP (p<0.001) levels compared to the control group. For GDF-15, it was observed an area under the ROC curve =0.825 (p<0.001, IC=0.722-0.927) ( Figure 2 ). FABP3 (r=0.344; p=0.046) and FABP4 (r=0.479; p=0.004) were positively correlated to BMI in the breast cancer group.

**Figure 1:** – Cardiovascular markers comparing breast cancer patients and control subjects. CXCL-16: C-X-C motif ligand 16; FABP3: fatty acid binding protein 3; FABP4: fatty acid binding protein 4; LIGHT: tumor necrosis factor superfamily member 14/TNFS14; GDF-15: Growth/differentiation factor 15; sCD4: soluble form of CD14; ucMGP: uncarboxylated Matrix Gla-Protein; BC: breast cancer patients; HS: healthy control subjects * Significant (p<0.05). ** Significant (p<0.001).

**Figure 2:** – ROC curve for GDF-15 levels considering the breast cancer patients treated with doxorubicin x control group.

Considering the breast cancer group, it is interesting to note that GDF-15 levels were higher in the triple negative group compared to other molecular groups (p=0.030), but this difference was non-significant after applying Bonferroni’s test ( Table 2 ). Accordingly, FABP3 levels were also higher in the group with high Framingham cardiovascular risk (p=0.022), but were non-significant after Bonferroni’s correction ( Table 3 ). No other cardiovascular marker showed differences in plasma levels according to the molecular type of tumor or cardiovascular risk.

**Table 2:** – Cardiovascular biomarkers according to the molecular subtype in breast cancer patients

Biomarkers	Luminal (12, 35.3%)	HER2+ (18; 52.9%)	Triple negative (4; 11.8%)	p-value
CXCL-16 (pg/mL)	1.35 [0.96 - 1.59]	1.52 [1.25 - 2.53]	1.38 [1.03 - 2.52]	0.370
FABP3 (pg/mL)	1.36 [1.15 - 12.89]	1.21 [0.92 - 1.80]	0.63 [0.09 - 15.26	0.444
FABP4 (pg/mL)	0.21 [0.06 - 0.67]	0.86 [0.14 - 1.32]	0.82 [0.10 - 17.05]	0.248
LIGHT (pg/mL)	0.06 [0.02 - 1.21]	0.86 [0.06 - 46.41]	0.42 [0.12 - 0.92]	0.203
GDF-15 (ng/mL)	11.38 [3.82 - 15.15]	14.75 [11.10 - 19.54]	24.79 [17.92 - 1899.90]	0.030 ^†^
sCD14 (pg/mL)	3.387 [0.026 - 5.574]	4.098 [1.362 - 5.031]	0.021 [0.005 - 4.767]	0.398
ucMGP (ng/mL)	0.09 [0.05 -18.93]	0.15 [0.09 - 0.29]	7.97 [0.06 - 15.87]	0.957

CXCL-16: C-X-C motif ligand 16; FABP3: fatty acid binding protein 3; FABP4: fatty acid binding protein 4 LIGHT: tumor necrosis factor superfamily member 14/TNFS14; GDF-15: Growth/differentiation factor 15; sCD4: soluble form of CD14; ucMGP: uncarboxylated Matrix Gla-Protein. ^†^ Non-significant after Bonferroni correction.

**Table 3:** – Cardiovascular biomarkers according to Framingham cardiovascular risk in breast cancer patients

Biomarkers	Low risk (n=15)	Intermediate risk (n=10)	High risk (n=9)	p-value
CXCL-16 (pg/mL)	1.34 [1.19 – 1.90]	1.40 [0.96 – 1.72]	2.32 [1.43 – 2.83]	0.101
FABP3 (pg/mL)	1.00 [0.59 – 1.27]	1.37 [1.14 – 12.21]	11.37 [1.06 – 19.54]	0.022 ^†^
FABP4 (pg/mL)	0.15 [0.08 – 0.62]	1.18 [0.48 – 1.41]	0.84 [0.08 – 1.41]	0.096
LIGHT (pg/mL)	0.41 [0.06 – 35.08]	0.06 [0.02 – 0.55]	4.35 [0.16 – 25.33]	0.115
GDF-15 (ng/mL)	11.74 [1.72 – 15.66]	14.91 [11.82 – 19.75]	17.53 [11.82 – 24.02]	0.066
sCD14 (pg/mL)	5.18 [3.51 – 6.78]	0.28 [0.02 – 5.23]	2.24 [0.47 – 4.52]	0.080
ucMGP (ng/mL)	0.16 [0.09 – 14.07]	0.88 [0.05 – 14.05]	0.11 [0.06 – 0.36]	0.620

CXCL-16: C-X-C motif ligand 16; FABP3: fatty acid binding protein 3; FABP4: fatty acid binding protein 4 LIGHT: tumor necrosis factor superfamily member 14/TNFS14; GDF-15: Growth/differentiation factor 15; sCD4: soluble form of CD14; ucMGP: uncarboxylated Matrix Gla-Protein. ^†^ Non-significant after Bonferroni correction.

## Discussion

The investigation, monitoring, and evaluation of cardiovascular injury in breast cancer patients under chemotherapy regimens have been widely studied. However, studies that include emerging biomarkers that are able to detect cardiovascular impairment in breast cancer patients under DOXO-based chemotherapy in advance, regardless of clinical cardiotoxicity, are rare. Therefore, the main findings of this study are: (i) breast cancer patients had higher GDF-15 levels, which showed good accuracy to differentiate this group and controls, according to the area under the ROC curve; (ii) breast cancer patients had lower levels of FABP3, FABP4, sCD14, and ucMGP; and (iii) there was a positive correlation between FABPs and BMI.

GDF-15 is a strong and independent predictor of CVD, cancer morbidity, and mortality in community-dwelling individuals. ^30^ The breast cancer group had GDF-15 levels that were 8.12-fold higher than in healthy subjects. In breast cancer, GDF-15 has been associated with metastasis and resistance toward trastuzumab. ^31^ GDF-15 increases due to several pathophysiological conditions; thus, elevated GDF-15 levels must be carefully interpreted. In this study, the reason(s) associated with increased GDF-15 levels in breast cancer patients remain unclear, since breast cancer biology and DOXO-chemotherapy are both conditions that can promote GDF-15 changes. GDF-15 is an emerging biomarker which is elevated in early subclinical disease and has prognostic utility for cardiovascular events and mortality. ^32^ Therefore, robust case-control studies that include at least one group of breast cancer patients treated with another class of chemotherapy medications could be useful to clarify this study’s hypothesis.

A prospective cohort by Demissei et al. ^6^ included 323 breast cancer patients who were treated with anthracycline- and/or trastuzumab-based regimens. In that study, no association between GDF-15, troponin, myeloperoxidase, and placental growth factor levels with changes in LVEF was found. Also, no changes in the GDF-15 levels were observed over the two years of study. In the baseline, GDF-15 levels in breast cancer patients who received DOXO-treatment were 704 [532–908] pg/mL and 599 [523–722] pg/mL for patients who received DOXO+Trastuzumab. In this study, GDF-15 was higher in triple negative patients, but the difference was not significant, which requires further studies with a larger population. In a multicenter cohort study, GDF-15 levels remained elevated even after 15 months of study in breast cancer patients (HER2+) under adjuvant therapy with an anthracycline-containing regimen followed by taxanes and trastuzumab. ^33^

FABP4 levels were lower in breast cancer patients than in controls in this study, which is an unexpected finding in agreement with Tsakogiannis et al. ^34^ FABP4 is highly expressed in adipocytes, but breast cancer patients did not show any differences in BMI compared to healthy controls. However, BMI is not the best method to evaluate adiposity; other markers of body fat composition should be applied to correlate with FABP4 levels. In fact, its levels showed a correlation with BMI in the breast cancer group in this study. Contrary to the observations included in this study, another case-control study found higher levels of FABP4 in breast cancer patients in contrast with healthy women and higher levels in luminal type breast cancer compared to HER2+/triple negative. However, they also suggest that BMI in breast cancer could be a factor affecting the expression of FABP4 because patients with breast cancer and a BMI ≥25 kg/m ^2^ presented higher levels of FABP4. ^34^ These data emphasize that FABPs are expressed by adipose tissue. Also, circulating FABP4 enhances the tumor stem cell-like phenotype via IL-6/STAT3/ALDH1-mediated activity, suggesting that circulating FABP4 released by host adipose tissue might trigger the exit from tumor dormancy ^35^ and that FABP-4 is up-regulated in certain subsets of macrophages in breast/mammary tumors, which enhances their ability to promote tumor growth and metastasis through IL-6-dependent pathways. ^36^

FABP3 levels were also lower in the breast cancer group compared to controls. It is known that FABP3 plays a key role in cardiomyocyte metabolism. However, it is possible to hypothesize that DOXO-chemotherapy could promote a decrease in FABP3 synthesis in cardiac tissue because DOXO induces cardiomyocyte apoptosis. ^37^ This was experimentally demonstrated by Sayed-Ahmed et al., ^38^ where the chronic use of DOXO resulted in a significant and dose-dependent decrease in FABP3 mRNA expression in cardiac tissue. Moreover, the loss of cellular lipid metabolism homeostasis due to reduced intracellular FABP3 content and impaired fatty acid supply seems to be a plausible hypothesis for the progression of heart failure and other CVDs. ^39^

Conway et al. demonstrated that the FABP3 gene promoter was hypermethylated and gene expression was reduced in breast cancer, indicating that FABP3 expression has an inhibitory effect on breast cancer. ^40^ On the other hand, FABP3 is a heart injury marker, since elevated levels are useful for the early diagnosis of acute myocardial infarction. ^41^ Plasma levels of FABP3 have already been investigated in the context of breast cancer chemotherapy. However, no differences were observed in those individuals who developed cardiotoxicity secondary to anthracyclines compared to individuals without onset cardiotoxicity. ^42^ Substantial experimental and clinical studies are necessary to clarify FABP3 behavior in this context. The positive correlation between FABP3 and FABP4 with BMI was already expected since these markers are directly associated with adipose tissue and lipid metabolism.

Although lower sCD14 levels were observed in breast cancer patients in this study, this is controversial, as some studies have shown that sCD14 levels were higher in patients with cancer than in patients with benign disease or healthy individuals. ^43 , 44^ The outcome of CD14 in inflammation is multifactorial, including the site of inflammation, CD14 expression level, characteristics of CD14 ligands, and competition between different CD14-dependent pathways. ^21^ CD14 is also expressed on the cell membranes of cardiomyocytes ^45^ and the apoptotic DOXO-effect on cardiomyocyte could reduce the soluble CD14. Also, sCD14 levels have also been determined in other studies as an acute phase reactant, ^46^ but the patients evaluated were not in the acute inflammatory phase, evaluated by C-reactive protein (CRP) measurements (data not shown). Therefore, these results should be interpreted cautiously and future studies in this context should be conducted in order to determine the role of sCD14 in this scenario.

Breast cancer patients also had lower ucMGP plasma levels compared to control subjects. According to Yoshimura et al., ^47^ the MGP gene is up-regulated in cases where the prognosis was poor, indicating that the mRNA levels of MGP are a potential prognostic indicator of breast cancer. However, no difference was observed in the protein expression by the tumor using immunohistochemistry. On the other hand, lower ucMGP levels were causally related to a decreased risk of coronary heart disease. ^48^ The inactive forms of MGP (like uc-MGP) are useful biomarkers of vitamin K deficiency, vascular calcification and CVD and may predict future risk of death or cardiovascular events. It is a vitamin K-dependent protein (VKDP), and it is released from cells into the bloodstream. Vitamin K attenuates inflammatory responses by blocking nuclear factor κB (NF-κB) signal transduction. Higher levels of ucMGP reflect the vascular calcification, which is one of the major risk factors for cardiovascular morbidity and mortality. ^49 , 50^ In this way, this study’s data suggest that DOXO-administration does not induce short-term cardiovascular calcification, which is an unlikely mechanism related to the cardiovascular toxicity of DOXO. The determination of vitamin K levels, such pro-inflammatory cytokines (like IL-6 and TNF-α; which accelerate the formation of VKDPs) and the quantification of other VKDPs, such as osteocalcin and growth arrest-specific 6 (Gas6) and Gla-rich protein (GRP), is strongly encouraged in future prospective clinical studies including patients with breast cancer under DOXO treatment.

### Limitations

This study presents limitations, such as a sectional study of a single center, conducted with patients who were undergoing chemotherapy only with DOXO. The small sample size is also an important limitation of this study. Moreover, as the biomarkers were not evaluated before the treatment, the cancer itself could influence some results. Consequently, further longitudinal studies for validation of these markers, with a huge population, should be conducted in order to evaluate their performance in monitoring cardiovascular changes caused by DOXO chemotherapy.

## Conclusion

The result of this study is a preliminary one, but it could contribute to a better understanding of the underlying mechanisms of doxorubicin-cardiotoxicity-based. Moreover, the findings suggest that GDF-15, FABP3, FABP4, sCD14, and ucMGP levels could be related to cardiovascular changes in breast cancer patients treated with DOXO. Further studies should be conducted in other populations in order to validate the results of this study.
